# Stinging Salvation: Harnessing Scorpion Venom Peptides for Revolutionary Pain Relief

**DOI:** 10.3390/toxins18030120

**Published:** 2026-02-26

**Authors:** Reza Mosaddeghi-Heris, Mojtaba Pandeh, Leila Ghorbi, Niloofar Taheri, Maedeh Shariat Zadeh, Kimia Bagheri, Paolo Martelletti

**Affiliations:** 1 Neurosciences Research Center (NSRC), Tabriz University of Medical Sciences, Tabriz 5165990001, Iran; 2 School of Medicine, Gonabad University of Medical Sciences, Gonabad 9691797852, Iran; 3 Department of Psychiatry and Behavioral Sciences, Stanford University, Palo Alto, CA 94305, USA; taheri@stanford.edu; 4 School of Medicine and Surgery, University of Naples Federico II, 80131 Naples, Italy; 5 Student Research Committee, Babol University of Medical Sciences, Babol 4717647745, Iran; 6 Tongji Medical College, Huazhong University of Science and Technology, Wuhan 430030, China; 7 School of Health, Unitelma Sapienza University, 00185 Rome, Italy

**Keywords:** scorpion venom peptides, non-opioid analgesics, ion channel blockers, neuropathic pain, anti-inflammatory

## Abstract

Peptides from scorpion venom, mainly in species such as *Olivierus martensii* (formerly *Olivierus martensii* Karsch, often designated BMK) (BmK) and *Tityus serrulatus* from the Buthidae family, show real promise as painkillers that skip opioids altogether. They work by hitting specific ion channels and dialing down inflammation. This review gathers information on their molecular setups: disulfide-bridged types and those without, weighing in at 3 to 10 kilodaltons (kDa). Structural features include motifs stabilized by cysteines. In pain signaling, they block voltage-gated sodium channels (NaV) such as NaV1.7 and NaV1.8; take the BmK analgesic–antitumor peptide (BmK-AGAP) for example. Additionally, scorpion venom heat-resistant peptide (SVHRP) reduces microglia activity. Tests on rodents using formalin injections, acetic acid writhing, and chronic constriction injury (CCI) setups reveal pain relief that depends on dose and stacks up to morphine. Pairings like AGAP with lidocaine decrease the effective dose by half. In terms of safety, therapeutic levels have low-toxicity with a median lethal dose (LD50) over 20 mg/kg. Issues crop up with immune responses, unintended targets, and differences in venom batches. Clinical information remains thin, so gaps persist. Engineered versions could change the game for neuropathic pain, inflammatory conditions, and cancer-related discomfort. Standardization plus Phase I studies would help move this forward.

## 1. Introduction

Chronic pain is one of the most significant health challenges across the world, and around 30 percent of adults are affected, with its prevalence increasing markedly with age [1,2,3]. It impairs daily functioning and leads to disability and a reduced quality of life in the context of various conditions such as chronic inflammatory diseases, neuropathic pain, and musculoskeletal disorders [2]. Opioids are known to be one of the most potent analgesics for acute and severe pain. However, when it comes to chronic pain, their long-term efficacy is limited [1,4]. Furthermore, several adverse effects involving the central nervous system, respiratory, and gastrointestinal systems, as well as the risks of tolerance, dependence, and misuse have been reported with opioids [5]. These limitations, alongside the ongoing opioid crisis, highlight an urgent need for effective non-opioid therapeutic approaches [1,6].

One important strategy used in non-opioid analgesic development is the modulation of peripheral ion channels that regulate nociceptive signaling [7,8]. Voltage-gated sodium channels, especially the NaV1.7 and NaV1.8, are central to both the initiation and propagation of action potentials in primary sensory neurons [9,10]. Human genetics and extensive preclinical studies have revealed these channels as compelling targets for pain treatment [11]. Small-molecule NaV1.7 inhibitors, however, have shown limited translation to clinical benefit [12]. The gap between target validation and therapeutic success has led to growing interest in naturally occurring peptides derived from venomous species, which have undergone millions of years of evolutionary refinement and frequently exhibit high potency and selectivity for specific ion channel subtypes [13,14].

A wide variety of bioactive compounds, such as disulfide-bridged peptides (DBPs) and non-disulfide-bridged peptides (NDBPs), are found in scorpion venoms, which serve as an example of this evolutionary optimization [15,16]. NDBPs have a broad range of biological roles, whereas DBPs often regulate NaV, KV, and CaV channel activity [16,17]. *Olivierus martensii* (formerly *Olivierus martensii* Karsch, often designated BMK) (BmK) is one of these peptides that has been used in traditional Chinese medicine for conditions involving pain and inflammation [18]. BmK AGAP is one of the most extensively studied peptides from this species and demonstrates potent analgesic activity through modulation of NaV channels, inhibition of Transient Receptor Potential Vanilloid 1 (TRPV1), and enhancement of local anesthetic effects [19]. Similarly, venom from *Tityus serrulatus* (Ts) is rich in α- and β-neurotoxins, which target the sodium and potassium channels [20]. Recent studies on engineered variants such as TsNTxP have reported promising anti-nociceptive and anti-inflammatory effects with reduced toxicity, highlighting their potential for therapeutic development [21].

In recent years, rapid progress has been made in characterizing the molecular diversity, structural mechanisms, and medicinal applications of scorpion venom peptides. High-resolution structural studies have provided new insights into NaV1.7 and NaV1.8 selectivity, supporting the rational engineering of more targeted and safer peptide analogs [22,23]. Additional research has revealed both peripheral anti-inflammatory properties and synergy with local anesthetics [19,24]. Together, these data support the potential of scorpion-derived peptides as innovative candidates for the treatment of chronic pain, including neuropathic pain and arthritis [24,25]. Important challenges, however, remain, including variability in venom composition, limited pharmacokinetic data, and the scarcity of clinical studies [22,24,25].

In this review, we discuss recent advances in scorpion venom peptides as an emerging class of non-opioid analgesics, as well as their molecular and structural diversity and key mechanistic pathways, with focus on the modulation of NaV1.7/1.8 and TRPV1. We also summarize preclinical evidence supporting their analgesic and anti-inflammatory properties. Moreover, we evaluate available safety data and the major challenges in assessing their translational potential. Finally, we discuss possible ways in which engineered peptide variants, recombinant production strategies, and standardized methodological approaches may accelerate the development of novel pharmacological preparations derived from scorpions for chronic pain.

## 2. Molecular Diversity and Structural Characteristics of Scorpion Venom Peptides

**Figure 1:**
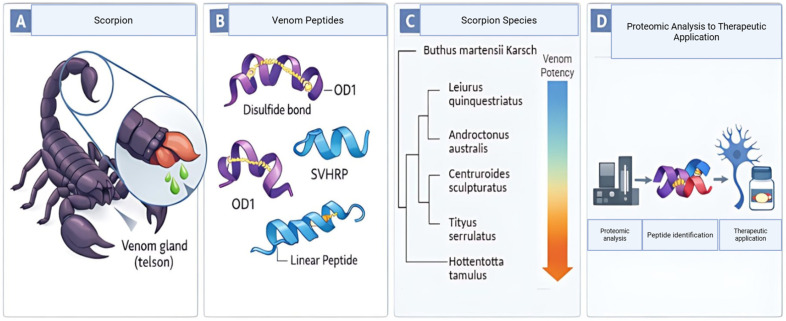
The evolution of scorpion venom peptides as non-opioid analgesics. (**A**) Where *Olivierus martensii* (formerly *Olivierus martensii* Karsch, often designated BMK) venom is collected, emphasizing the telson gland. (**B**) representations of certain analgesic peptides (AGAP, BmK I, OD1, SVHRP, TSNTXP), in three dimensions that show their tertiary folds. The coloring scheme reflects each peptide’s functional class and highlights disulfide bridges. (**C**) Analgesic potency and evolutionary variation within Buthidae species. (**D**) The progression from proteomic analysis of crude venom to the rational design of potential non-opioid drug candidates.

Scorpion venoms are complex mixtures of metal ions, biogenic amines such as serotonin, small organic molecules, and enzymes, including hyaluronidase, metalloproteinases, and phospholipases A_2_ [26]. They also contain numerous low–molecular-mass peptides and neurotoxins that account for most of their pharmacological activities [17]. Peptides are the main medically relevant fraction and are usually divided into disulfide-bridged peptides (DBPs) and non-disulfide-bridged peptides (NDBPs) on structural and functional grounds [22]. DBPs typically comprise 13–70 amino acids stabilized by three or four disulfide bonds and form compact scaffolds that modulate Na^+^, K^+^, Ca^2+^, Cl^−^ or TRP family channels [24,27,28]. Many of these toxins are classified as NaTx, KTx, CaTx, ClTx or TRPTx according to their primary ion-channel target [27,29]. NDBPs usually contain 13–56 residues and lack disulfide bridges [24,27]. Members of this group display diverse activities, including antimicrobial, anti-inflammatory, immunomodulatory, analgesic, antioxidant and antiviral effects [24]. Across both families, most mature scorpion peptides fall within a 3–10 kDa mass range, consistent with their short length and compact folds [22,30]. An overview of scorpion venom peptide extraction, representative analgesic peptide scaffolds, and evolutionary/proteomic context is shown in Figure 1.

Low–molecular-mass neurotoxins are especially abundant in scorpion venoms and are largely responsible for their acute toxicity in mammals [26]. Buthidae is the largest and best-studied scorpion family and includes medically important genera such as Androctonus, Buthus, Mesobuthus, Hottentotta, Parabuthus, Tityus, Centruroides and Leiurus [31,32,33]. Severe human envenomings worldwide are dominated by buthid species, whose venoms are enriched in low-molecular-mass neurotoxins that target ion channels [30,31]. Proteomic work on African Buthidae confirms that these venoms contain abundant Na^+^- and K^+^-channel toxins together with peptides that display anticancer and other bioactivities [30]. Many analgesic peptides characterized to date have been isolated from the Chinese scorpion *B. martensii*, which has a long history of use in traditional medicine [17]. The long-chain Na^+^-channel toxin BmK AGAP from *B. martensii* reduces somatic and visceral pain in experimental models and also inhibits proliferation, migration and epithelial–mesenchymal transition in several cancer cell types [34].

IMe-AGAP, an AGAP-like peptide from *Mesobuthus eupeus*, shares the same length (66 residues) and exerts comparable antitumour-analgesic effects [35]. Recent venomics studies have expanded the known toxin diversity of Buthidae. A dual proteomic and functional analysis of several African buthid venoms revealed rich repertoires of Na^+^- and K^+^-channel toxins, antimicrobial peptides and enzymes such as peptidases [30]. Venomic work on the New World scorpion *Ananteris platnicki* uncovered additional Na^+^- and K^+^-channel toxins, antimicrobial peptides (AMPs) and many sequences with no clear homology to known proteins [36]. A combined venom-gland transcriptomic and proteomic study of the Hentz striped scorpion *Centruroides hentzi* showed similarly high toxin diversity in a species that is essentially harmless to humans [37]. Modern venom research routinely integrates high-throughput transcriptomics, mass-spectrometric proteomics and database-driven annotation, with scorpion-specific resources such as ScorpDb providing curated toxin sequences and metadata, to map venom composition at the peptide level [22,38]. The venom-gland transcriptome and proteome of the sea snake *Hydrophis curtus*, for example, were characterized using next-generation sequencing and LC–MS/MS workflows [39]. The transcriptome of the tarantula *Pamphobeteus verdolaga* was re-annotated to discover novel bioactive peptides using similar approaches [40].

### 2.1. Classification and Sources

From the perspective of pain and inflammation, scorpion venom peptides can be grouped into two overlapping functional classes [17,22,41]. The first comprises neurotoxic channel-modulating peptides, most of which are DBPs that affect voltage-gated Na^+^, K^+^, Ca^2+^ or Cl^−^ channels [17,24,41]. The second comprises antimicrobial and immune-modulatory peptides, many of which are NDBPs or short antimicrobial peptides (ssAMPs) and some of which also display dual antimicrobial–analgesic or antitumor–analgesic actions [27]. Neurotoxic channel-modulating peptides alter the excitability of peripheral and central neurons by binding to Na^+^-, K^+^-, Ca^2+^- and Cl^−^-channel subtypes [4,41]. In clinical envenoming, activation of voltage-gated Na^+^ channels by scorpion toxins is the principal driver of intense pain. Additional pronociceptive mechanisms include inhibition of selected K^+^ channels, activation of TRPV1 channels and the release of pro-inflammatory mediators such as IFN-γ, IL-1β and TNF-α [41,42]. Despite their overall pronociceptive effect, scorpion venoms also contain peptides with analgesic activity [17]. Fewer analgesic molecules have been identified and pharmacologically characterized than pain-inducing toxins, but the available data indicate several distinct mechanisms, including blockade of specific Na^+^ channels and modulation of inflammatory pathways [41,43]. Among the best-characterized examples, BmK AGAP from *B. martensii* and IMe-AGAP from Iranian M. eupeus each comprise 66 amino acids and strongly inhibit somatic and visceral pain in animal models [34,35]. BmK AGAP additionally inhibits proliferation and migration and induces apoptosis in breast and other cancer cell lines, suggesting potential as a dual antitumour–analgesic lead [34]. IMe-AGAP shares structural similarity with BmK AGAP and has been proposed as another antitumour-analgesic candidate [35]. Site-directed mutagenesis studies on BmK AGAP show that the Cys16–Cys36 and Cys22–Cys46 disulfide bonds and residues Gly36, Arg37, Trp57 and Asn63 form a pharmacophore domain important for analgesic activity [35]. Antimicrobial and dual-function peptides in scorpion venoms are mostly NDBPs or ssAMPs [27]. These peptides are typically 13–20 residues long, lack disulfide bridges and are often C-terminally amidated [44]. IsCT, a 13-residue amidated peptide from Opisthacanthus madagascariensis, was one of the first scorpion ssAMPs to be characterized and shows potent activity against Gram-positive and Gram-negative bacteria with relatively low haemolysis [44]. Scorpion AMPs are generally amphipathic and positively charged and can be divided into three main structural categories: cysteine-containing peptides with disulfide bridges, amphipathic α-helical peptides without cysteine and glycine- or proline-rich peptides [17,27]. Studies on stigmurin from Tityus stigmurus and its analogs StigA6, StigA16, StigA25 and StigA31 demonstrate that increasing net positive charge and hydrophobicity can enhance antibacterial potency and broaden the spectrum of activity [27]. Additional AMPs such as Pantinins, TsAP-2, Marcin-18 and the North African peptides AaeAP1 and AaeAP2 further extend this NDBP-derived AMP repertoire [27]. Several of these antimicrobial peptides also influence inflammatory signaling or tumour cell survival, creating antimicrobial–immunomodulatory–analgesic hybrids with promising translational potential [22]. Geographically, Buthidae is the dominant scorpion family in Africa and large parts of Asia, with medically important species concentrated in North Africa, the Middle East, India and China [31]. Taxonomic work has shown that this family encompasses genera such as Androctonus, Buthus, Leiurus, Mesobuthus, Parabuthus, Hottentotta, Tityus and Centruroides [31,32,33].

Venom-based studies from southern and northern Africa emphasize that Buthidae venoms in these regions combine high toxin diversity with significant anticancer potential [30]. Asian buthid species, particularly *B. martensii* and various Mesobuthus scorpions, have yielded many of the best-characterized analgesic peptides [24]. New World buthids such as *Ananteris platnicki* and *Centruroides hentzi* further broaden this repertoire, as venomics and venom-gland transcriptomics reveal abundant Na^+^- and K^+^-channel toxins, AMPs, peptidases and numerous unclassified venom proteins even in species that pose little or no threat to humans [36,37].

### 2.2. Structural Features

Disulfide-bridged peptides (DBPs). DBPs in scorpion venom generally comprise 13–70 amino acids and contain three or four disulfide bonds that stabilize compact peptide scaffolds [24,27]. Many DBPs adopt cysteine-stabilized α/β folds and act as potent modulators of Na^+^, K^+^, Ca^2+^ or Cl^−^ channels [29]. On the basis of length and target selectivity, DBPs can be subdivided into short-chain toxins of about 30–40 residues with three or four disulfide bridges, which predominantly act on K^+^-, Cl^−^- and Ca^2+^-channels, and long-chain toxins of about 60–70 residues with four disulfide bridges, which mainly target Na^+^ channels [24]. Long-chain Na^+^-channel toxins are further classified as α- or β-scorpion toxins according to their binding sites and electrophysiological effects on voltage-gated sodium channels [24]. α-Toxins bind to site 3 of the channel and slow or inhibit deactivation, whereas β-toxins bind to site 4, typically shifting activation towards more hyperpolarised potentials and reducing peak current amplitude [24]. Site-directed mutagenesis of BmK AGAP has identified two disulfide bonds (Cys16–Cys36 and Cys22–Cys46) and residues Gly36, Arg37, Trp57, and Asn63. These features are critical determinants of its analgesic activity [35].

Non–disulfide-bridged peptides (NDBPs). NDBPs usually contain 13–56 amino-acid residues and lack disulfide bonds [24,27]. Many NDBPs form amphipathic α-helices at membrane interfaces, whereas glycine- or proline-rich sequences tend to adopt more flexible conformations [27]. Their positive net charge and hydrophobic surfaces promote interaction with and disruption of microbial membranes, accounting for classical AMP activity [27]. C-terminal amidation, which is common among ssAMPs, further modulates peptide stability, selectivity and potency [44]. Short antimicrobial peptides within this disulfide-free group typically comprise 13–20 residues, as exemplified by IsCT from O. madagascariensis [44]. IsCT combines low haemolytic activity with robust antibacterial activity against both Gram-positive and Gram-negative bacteria [44]. As shown for stigmurin and analogs such as StigA6, StigA16, StigA25 and StigA31, modest sequence changes that increase net charge and hydrophobicity can significantly enhance antimicrobial potency and broaden the antibacterial spectrum. Notably, several NDBPs exhibit dual antimicrobial–analgesic activity, providing a structural basis for the multifunctional peptides discussed in later sections [17,22,24,41]. Pantinins, TsAP-2, Marcin-18 and AaeAP1/2 illustrate additional NDBP-derived AMP scaffolds with promising pharmacological profiles [27].

Analytical characterization. Since the mid-twentieth century, advances in purification and structural analysis have progressively revealed the diversity of scorpion venom peptides [17]. Early biochemical work, including the isolation of 11 peptide neurotoxins from *Androctonus australis*, *Buthus occitanus tunetanus* and *Leiurus quinquestriatus*, established the basic structural features and ion-channel targets of many DBPs [17]. Subsequently, the discovery of AEP, a 61-residue peptide with antiepileptic activity from *Olivierus martensii* (formerly *Olivierus martensii* Karsch, often designated BMK), provided further evidence for the therapeutic potential of scorpion peptides [17]. In recent years, high-throughput sequencing of venom-gland transcriptomes combined with LC–MS/MS-based proteomics has been widely applied across venomous taxa [22]. For instance, the venom-gland transcriptome and proteome of *Hydrophis curtus* were characterized using next-generation sequencing and complementary proteomic strategies, revealing numerous toxin families [39]. A similar combination of methods was used to re-annotate the venom-gland transcriptome of *Pamphobeteus verdolaga* to prospect for novel bioactive peptides [40]. Venom-gland transcriptomics and proteomics of *Centruroides hentzi* likewise demonstrated high toxin diversity and identified many peptides whose functions remain unknown [37]. These integrative omics datasets underpin the structural and taxonomic framework used to select DBPs and NDBPs as templates for next-generation analgesic and multifunctional therapeutics [22].

## 3. Mechanisms of Scorpion Venom Peptides in Pain Pathways

Scorpion venom peptides, traditionally viewed solely as toxins, that immobilize or kill prey, are now considered important candidates for drug development and design [22]. Venoms from diverse animals are rich in peptide toxins with high specificity and potency for molecular targets and have become a valuable source for drug discovery [45]. Scorpion venom peptides have also been explored as anticancer agents that selectively target cancer cells, inhibit tumor proliferation, and modulate immune responses [46].

In pain, scorpion venom peptides mainly modulate ion channels that control nociceptor excitability and neurotransmission, and several also exert anti-inflammatory and neuroprotective actions through effects on immune cells and glia [47].

### 3.1. Ion Channel Modulation

Disulfide-bridged peptides (DBPs) are the main scorpion venom components with neurotoxic effects; they contain three to four disulfide bridges and mostly affect membrane ion channels in excitable and non-excitable cells [17]. α-type sodium channel toxins (α-NaTx) and β-type sodium channel toxins (β-NaTx) target sodium channels via distinct mechanisms: α-NaTx prolong channel opening, whereas β-NaTx lower activation threshold, leading to persistent overexcitation and pain [41].

In small-diameter dorsal root ganglion (DRG) neurons, NaV1.8 channels intrinsically generate tetrodotoxin-resistant resurgent sodium currents that activate at relatively depolarized potentials, indicating that NaV1.8 can underlie the slow resurgent currents previously described in nociceptive neurons. Gain-of-function disease mutations such as the mouse T790A “Possum” mutation and the human small fiber neuropathy mutation G1662S markedly amplify NaV1.8 resurgent currents by slowing inactivation and increasing overlap between activation and inactivation curves, without substantially changing peak current density [48].

These mutations greatly increase spontaneous firing, reduce current threshold, broaden action potentials, and promote multiple early afterdepolarizations in DRG neurons, whereas knockdown of the NaVβ4 subunit selectively reduces NaV1.8 resurgent current and normalizes firing. Together, these data indicate that enhanced NaV1.8-mediated resurgent currents via Navβ4-dependent open-channel block are a major driver of nociceptor hyperexcitability and strongly support a mechanistic link between NaV1.8 gain-of-function mutations, aberrant sensory neuron firing, and neuropathic pain phenotypes such as painful small fiber neuropathy [48].

In trigeminal ganglion (TG) neurons, the tetrodotoxin-resistant sodium channels NaV1.8 and NaV1.9 are selectively expressed in small- and medium-diameter pain-sensing neurons, where NaV1.8 contributes to the rising phase of the action potential, and NaV1.9 regulates resting membrane potential and subthreshold depolarizing responses. In the infraorbital nerve chronic constriction injury (IoN-CCI) model of trigeminal neuralgia, TG NaV1.8 expression and current density are reduced. The steady-state activation and inactivation curves are shifted in directions interpreted as promoting abnormal discharge and increased excitability, contributing to mechanical allodynia, thermal hyperalgesia, and spontaneous facial grooming [49]. Systemic administration of the β-type scorpion toxin Syb-prII-1, which blocks NaV1.8 with nanomolar potency, further decreases NaV1.8 protein levels and current density, reverses IoN-CCI–induced gating abnormalities, and produces robust, dose-dependent analgesia in IoN-CCI rats, with high doses achieving effects comparable to morphine without motor impairment; Syb-prII-1 also down-regulates phosphorylation of ERK1/2, JNK, p38, ERK5, and CREB in trigeminal tissue [49].

BmK I directly targets NaV1.8 to enhance nociceptor excitability, dose-dependently increasing NaV1.8 current in small DRG neurons, inhibiting fast and slow inactivation, and shifting activation and steady-state inactivation curves in a hyperpolarized direction, thereby reducing the threshold for excitability and increasing action potential firing. Intraplantar BmK I increases NaV1.8 mRNA and protein expression and TTX-R Nav1.8 current density in DRG neurons, and BmK I–induced spontaneous pain and mechanical allodynia, including in complete Freund’s adjuvant–inflamed rats, are significantly alleviated by the NaV1.8 blocker A-803467 or NaV1.8 knockdown with antisense oligodeoxynucleotides [50].

Voltage-gated calcium (Cav) channels regulate excitability and transmitter release in nociceptive pathways; among them, N-type Cav2.2, R-type Cav2.3, and T-type Cav3.2 channels have been implicated in neuropathic and inflammatory pain, with Cav3.2 highly expressed in nociceptors [51]. Scorpion peptides provide pharmacological tools for these channels: kurtoxin from *Parabuthus transvaalicus* is structurally related to α-scorpion NaV toxins and acts as a relatively selective inhibitor and gating modifier of low-threshold Cav3.1 and Cav3.2 channels, with weaker effects on Cav2.2 and NaV currents, although its impact on pain behavior has not yet been evaluated in vivo. Scorpion toxins also modulate potassium and other ion channels involved in pain. Hakim et al. demonstrated that the peptide BmP01 from the scorpion *Mesobuthus martensii* induces pain similarly to capsaicin, with diminished effects on Kv1.3 but potentiated effects on TRPV1 under acidic conditions [52]. γ-KTx peptides, containing 3 or 4 disulfide bridges, can selectively block Kv11.x (hERG) channels through high-affinity targeting by their turret regions, whereas κ-KTx, which have two disulfide bridges and a CSαα scaffold of two short α-helices connected by a β-turn, display low-affinity potassium channel inhibition. The scorpion toxin AGAP (anti-tumor analgesic peptide) potently inhibits HV1 currents, and an AGAP mutant with reduced NaV channel activity but intact HV1 activity (AGAP/W38F) has been described [51].

### 3.2. Anti-Inflammatory and Neuroprotective Roles

In acetic acid–writhing, carrageenan-induced rat paw swelling, xylene-induced mouse ear swelling, and IoN-CCI models, the scorpion toxins DKK-SP1 and DKK-SP2 show antiinflammatory and analgesic activity by inhibiting NaV channels [18]. DKK-SP1 reduces NaV1.8 expression and current in DRG neurons and decreases pro-inflammatory cytokines COX-2 and IL-6 while elevating anti-inflammatory IL-10, whereas DKK-SP2 inhibits NaV1.7 expression and current in hNaV1.7-CHO cells and displays significant analgesic effects [18].

Many scorpions K^+^ channel–acting toxins (KTx) target Kv1.3, which is an attractive pharmacological target for autoimmune diseases and is highly expressed on effector memory T cells; Kv1.3 maintains the negative membrane potential that supports Ca^2+^ influx, NFAT activation, IL2 production, and T-cell proliferation [53]. Several Kv1.3-blocking peptides, including Vm24, HsTX1 and its analog HsTX1[R14A]/PEG-HsTX1[R14A], ImKTx88, Ts6, and Ts15, have shown in vivo efficacy in rodent models by reducing delayed-type hypersensitivity reactions, attenuating inflammation in arthritis, and improving disease severity and blood–brain barrier integrity in experimental autoimmune encephalomyelitis, supporting scorpion Kv1.3 blockers as leads for TEM cell–targeted immunosuppressive therapies [53].

Scorpion venom heat-resistant peptide (SVHRP) attenuates LPS-induced neuroinflammation by reducing microglial activation and pro-inflammatory mediator production in vivo and in vitro, at least partly via inhibition of NF-κB and MAPK signaling, including decreased nuclear p65 and reduced phosphorylation of p38 and JNK [54]. Because these effects have so far been demonstrated mainly in acute LPS-stimulation paradigms rather than chronic neuropathic or neurodegenerative pain models [54], they may overestimate the anti-inflammatory potency of SVHRP, and additional chronic in vivo models will be required to clarify its translational relevance.

SVHRP is a low-toxicity, heat-stable polypeptide from *Olivierus martensii* (formerly *Olivierus martensii* Karsch, often designated BMK) with high purity, properties that favor its use in medical research [55]. A synthetic derivative, SVHRSP, protects dopaminergic neurons by inhibiting NOX2 activation, blocking p47phox membrane translocation, and weakening microglial activation and M1 polarization in experimental models of Parkinson’s disease [56]. SVHRSP attenuates PM_2.5_-induced microglial M1 polarization and suppresses cytotoxic inflammatory mediators via a TLR4-mediated autophagy/PI3K/AKT/NF-κB signaling pathway [57]. In addition, SVHRSP alleviates PM_2.5_-induced mitochondrial dynamics imbalance and neuroinflammation by downregulating the PGC-1α/SIRT3 signaling pathway [58].

**Figure 2:**
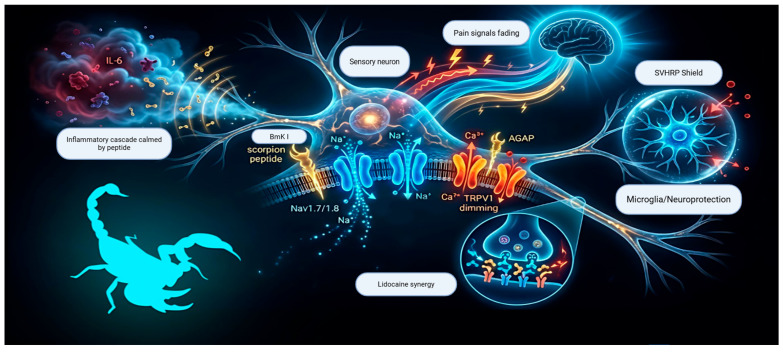
Mechanism of action of analgesic scorpion venom peptides. The multi-target regulation of pain pathways in a central neuron by certain peptides is shown in this schematic, which results in reduced inflammation and nociceptive signaling. Important interactions include cytokine suppression with microglial attenuation, TRPV1 inhibition with synergistic lidocaine effects, and sodium channel blocking (NaV1.7/1.8).

Whole-cell patch-clamp recordings showed that AGAP, in addition to its HV1-modulating actions described above, inhibited TRPV1 and increased the analgesic effect of lidocaine and inhibited KCNQ2/3 currents; this possible synergistic interaction suggests a potential approach for optimizing postoperative analgesia [19]. These multi-target analgesic mechanisms are summarized in Figure 2. Table 1, Table 2 and Table 3 summarize the main scorpion venom peptides covered in this review, their primary targets, and the pain models in which they have been evaluated.

## 4. Preclinical and Clinical Evidence

Preclinical studies show that scorpion venom-derived peptides are promising non-opioid analgesics. Preclinical testing in rodents consistently reveals dose-dependent antinociception [19,51]. These peptides target pain-related ion channels, such as voltage-gated sodium channels (NaV) and transient receptor potential channels (TRP) [25,63,64]. Clinical evidence from formal trials on scorpion venom peptides remains limited, despite a growing preclinical dataset. Most clinical observations come from traditional uses, with no randomized controlled trials on purified peptides for pain management. Human-related findings are mainly historical or anecdotal reports involving diluted crude venoms. In several endemic regions, processed or diluted scorpion venom has historically been used in traditional medicine for the management of scorpion stings. However, clinical application is limited by residual toxicity and poor biological stability [65]. Venom-derived peptides may match or exceed conventional opioids in potency and offer a mechanism-based advantage by avoiding opioid receptor side effects [51,66].

Controlled human trials are almost absent, and pharmacokinetics, safety, and formulation remain unresolved issues. These challenges limit clinical translation. Scorpion venom peptides should therefore be regarded as promising experimental analgesics, not approved medications.

### 4.1. Preclinical Studies

Various rodent models have been used to evaluate scorpion venom peptides for their preclinical effectiveness, including the acetic acid writhing test and the formalin test, which model visceral pain and inflammation, respectively [65,67,68,69]. The acetic acid writhing test is a sensitive model of peripheral visceral pain based on chemically induced abdominal constrictions, while the formalin test evaluates both acute (Phase I) and inflammatory (Phase II) nociception through biphasic paw responses. Together, these complementary models allow simultaneous assessment of peripheral and central components of analgesia [51,59,70,71]. Representative preclinical efficacy studies are summarized in Table 4.

There is evidence that some toxins extracted from BmK (*Olivierus martensii* (formerly *Olivierus martensii* Karsch, often designated BMK)) scorpion venom were equally effective as morphine in mice and rats [51,66,72]. For example, the peptide Syb-prII-1 (4.0 mg/kg in rats) displayed an analgesic effect similar to that of morphine in a chronic infraorbital neuralgia model, dose-dependently inhibiting NaV1.8 channels [66]. Makatoxin-3, administered at a dose of 450 nmol/kg in mice, elicited potent analgesia in models of inflammatory pain, such as in the formalin test and the complete Freund’s adjuvant-induced mechanical pain [61]. Other peptides derived from BmK, such as BmK IT-AP, BmK AngP1, BmK AS, and BmK IT2, have exhibited analgesic action in the acetic acid, formalin, and thermal pain tests. Thus, this underscores the great antinociceptive potential of the various BmK venom components. While rodent models do show promise, translational gaps exist because species differ in how ion channels (such as NaV channels) are expressed and how pain is processed, which makes it difficult to directly extrapolate their effectiveness to human pain. Describes the need for further analysis and criticality of translational gaps [62,73,74,75].

When BmK AGAP was co-administered with lidocaine in a CCI rat model, it potentiated analgesic effects and increased analgesia duration in a dose-dependent manner. Specifically, AGAP at 25, 50, or 100 μg/kg increased Paw Withdrawal Threshold (PWT) and duration of analgesia compared to either drug alone [76,77]. BmK AGAP inhibits TRPV1 and KCNQ2/3 currents, and its analgesic effects are tied to the inhibition of spinal MAPK signaling, particularly via p-MAPK-dependent mechanisms, with a weak inhibition of NaV1.7 current [78,79].

Makatoxin-3 (MkTxs) has shown strong pain-relieving effects in inflammatory pain models, such as the formalin test and CFA-induced pain, with analgesic action that is both non-narcotic and independent of opioids [66,80,81]. Another neurotoxin, Syb-prII-1 from *Olivierus martensii* (formerly *Olivierus martensii* Karsch, often designated BMK), was tested in animal models of trigeminal neuralgia and found to significantly reduce pain behaviors without causing motor impairment, even at higher doses [49,82]. Its effects are comparable to morphine and are mediated through the modulation of NaV1.8 channels and the suppression of MAPK signaling pathways.

TsNTxP (Tityus serrulatus Non-Toxic Protein), a non-toxic protein isolated from *Tityus serrulatus* venom, was assessed in Swiss mice and shown to have the ability to raise the pain threshold for acute and neuropathic pain [21]. It produced significant pain relief in tail-flick and capsaicin-induced nociception tests, with effects comparable to carbamazepine. TsNTxP also reduced sensitivity to mechanical and cold stimuli in neuropathic pain models. Its antinociceptive action comes from suppressing glutamate release in the spinal cord, rather than targeting voltage-gated sodium channels like typical neurotoxins, and it does not cause toxic side effects [83,84].

### 4.2. Emerging Clinical Insights

Despite extensive and compelling preclinical evidence, no scorpion venom-derived peptide has yet progressed into randomized human analgesic trials. A synthesis of contemporary reviews reveals no randomized controlled trials of purified scorpion peptides for human analgesia [85,86].

The observations made by humans can be classified into two informal categories: (i) ethnopharmacological/traditional uses (e.g., topical scorpion preparations used in regional folk medicine) and (ii) case reports or clinical series addressing scorpion envenomation and management [85]. Occasionally, these sources report analgesic or symptomatic effects after local application of scorpion material, but they lack standardized dosing, PK/PD characterization, and objective outcome measures necessary for clinical testing. As a result, anecdotal or ethnopharmacological evidence must always be interpreted with caution. It cannot be used in place of controlled clinical trials because it lacks standardization of dosing and has inherent reporting bias. Given the near-absence of scorpion-peptide human trials, useful translational analogs come from other venom-derived peptides that have successfully reached clinical practice [87]. Furthermore, anecdotal reports, may be influenced by cultural and reporting biases, further underscoring the necessity of rigorous, controlled clinical trials.

Ziconotide (ω-conotoxin MVIIA), an N-type Ca^2+^ channel blocker approved for intrathecal refractory pain, exemplifies both the promise and the pitfalls of venom-derived therapeutics: marked efficacy, requirement for intrathecal delivery [88], a narrow therapeutic window, and notable neuropsychiatric adverse effects, all of which inform first-in-human study design, dosing strategies, and safety monitoring [88,89,90]. Beyond Ziconotide, the regulatory approval of crofelemer, an antidiarrheal drug derived from the Croton lechleri tree (often confused with a venom source in folk medicine), further illustrates that successful translation of natural products, even with non-traditional mechanisms, is achievable if formulation and delivery challenges are overcome [91]. Unlike ziconotide, which is limited by central nervous system toxicity and neuropsychiatric adverse effects, scorpion-derived peptides such as BmK-AGAP demonstrate predominantly peripheral mechanisms and favorable safety profiles in preclinical models.

Developing scorpion peptide therapies requires improving stability, bioavailability, reducing immune reactions, and minimizing neurotoxicity. Several strategies can be used to overcome these barriers, including stabilized analogs, PEGylation, and nanoparticles or depots. Early clinical trials should focus on safety, toxicology, and immunogenicity assessments if systemic use is unsafe. A small-molecule NaV1.7 inhibitor such as Pfizer’s PF-05089771, though promising preclinically, has not shown significant pain relief in clinical trials [92,93,94].

After early-phase clinical trials failed to meet efficacy endpoints, vixotrigine (CNV1014802) and the acylsulfonamide inhibitors GDC-0276 and GDC-0310 were also discontinued. Clinical attention has been shifted to NaV1.8 as a more promising peripheral analgesic target following these failures. A NaV1.8 inhibitor, VX-548 (suzetrigine), was shown to reduce acute pain, but mixed results have been reported in phase II sciatica studies, tempering expectations for broad use [94,95,96,97]. 

It is clear that BmK-AGAP and TsNTxP exemplify complementary translational leads in inflammatory and neuropathic rodent models. BmK-AGAP exhibits dose-dependent efficacy by inhibiting partial NaV, TRPV1, and KCNQ2/3, modulating spinal MAPK signaling, and exhibiting synergistic effects with local anesthetics. TsNTxP exerts robust antinociception predominantly by suppressing presynaptic glutamate release, representing a non-NaV mechanism potentially advantageous in central-sensitization states [60,86,98].

Taken together, these results place scorpion peptides on the shortlist of promising but unproven candidates ready for translation into clinical therapy, and their success will depend on the results of properly designed early-phase trials.

## 5. Safety Profile and Challenges

Preclinical pain studies have demonstrated a generally good safety profile for peptides produced from scorpion venom, particularly when administered at therapeutic quantities isolated from the toxic components of crude venom. Recent reviews have shown that several venom peptides have analgesic or anti-inflammatory effects in vivo with little to no apparent toxicity, such as no organ damage or discernible behavioral side effects when given within defined dosage ranges [17]. Buthicyclin, a venom-derived peptide with notable analgesic effects without causing acute toxic effects (LD_20_ > 20 mg/kg), was shown to be a particularly interesting therapeutic option in the 2025 study [99]. These findings suggest that, with appropriate isolation and dosing protocols, venom-derived analgesic peptides may achieve a clinically relevant therapeutic window.

Peptide treatments derived from venom sources may contain immunogenic epitopes that might cause hypersensitivity reactions or neutralizing antibody responses, especially if the sequences are non-human or given often. Because immunogenicity can reduce therapeutic efficiency or cause immune-mediated adverse events in clinical settings, it is crucial to screen toxin-derived peptides for possible B lymphocyte and T-cell epitopes during early development, according to both computational and experimental immunology studies [12,100,101]. Scorpion venom can damage non-target tissue due to interaction with homologous ion channels expressed in cardiac or central nervous system tissues, which is a significant safety concern [102,103].

The composition of scorpion venom is not always consistent. It is a variety resulting from seasonal changes, differences in species (such as age, sex, size, and geographic origin), and how it is collected and processed, which include electrical stimulation parameters and post-harvest processing. This inconsistency renders the venom unreliable for research and potentially dangerous for medical use. To create a safe, mass-producible drug, scientists must carefully verify its chemical structure and test its strength and purity at every step, from raw venom to a lab-made version. Recent scientific investigation confirms the remarkable inconsistency of the venom. Additionally, strict and reliable methods are necessary to identify, measure, and control the quality of its components [26,104,105,106]. Finding venom’s active components requires sophisticated separation and analysis techniques. However, standardized workflows for collection, fractionation, and batch release remain largely unavailable. Without harmonized extraction and analytical standards, batch-to-batch differences in peptide isoforms, post-translational modifications and folding state will confound safety and efficacy comparisons across studies and impede regulatory approval [107,108].

These challenges, although substantial, are not insurmountable, as demonstrated by the successful clinical translation of ziconotide, a cone snail venom-derived peptide now approved for the treatment of severe chronic pain.

### 5.1. Adverse Effects of Scorpion Venom

Depending on the dosage and mode of exposure, scorpion venom can cause both systemic multi-organ effects and local nociceptive/inflammatory reactions. *Tityus serrulatus* venom injection caused severe mechanical and thermal hyperalgesia in a mouse model, along with dose-dependent spontaneous pain-like behavior (licking, flinching of the paws) [108]. Additionally, this local venom injection increased levels of pro-inflammatory cytokines (such as TNF-α and IL-1β) and immune cell infiltration (macrophages and neutrophils) into the affected tissue, suggesting that local pain is at least partially mediated through neuro-immune activation and inflammation rather than purely neurotoxic effects [109]. These findings illustrate how venom peptides/proteins can cause local tissue effects that include nociception and inflammation, even without systemic spread.

Moreover, scorpion venom peptides can induce severe systemic symptoms, such as tachycardia, diaphoresis, profuse sweating, psychomotor agitation, tremors, nausea, vomiting, sialorrhea, and either hypertension or hypotension [109]. For instance, 24 h after envenomation, rats given whole venom from *Leiurus macroctenus* revealed large reductions in the total protein content of several important organs (~16.4% drop in brain protein, ~14.7% reduction in liver). Middle-mass molecules (MMMs) measured at absorbances of 210 nm and 254 nm in all evaluated organs showed a significant increase in the same research. MMMs indicate extensive cellular and extracellular disturbance throughout organ systems and are thought to be indicators of endogenous intoxication and tissue damage [110]. Another investigation indicated that the activity of certain destructive enzymes, which are named proteases, dramatically increased in several organs, such as the heart and lungs, in response to scorpion venom [111]. When rats were exposed to the *L. macroctenus* venom, the levels of immune and growth signals in their lung tissue were changed. After 24 h, there were increased amounts of anti-inflammatory molecules (IL-4, IL-10), interferon-γ, key regulators like HIF-1α and NF-κB, and growth factors, which include FGF-2, VEGF, and EGF. This combination of changes shows that the venom triggers a complex immune response [112].

### 5.2. Overcoming Production Barriers

The difficulties of working with natural venoms, including their inconsistent composition, limited supply, and complex molecular structures, have led to the development of two main technical approaches: synthetic peptide engineering and recombinant manufacture. The first method, known as recombinant expression, allows for the mass production of venom peptides while preserving their structure. Advances in synthetic biology indicate that laboratories can now produce these peptides in a controlled environment by using different cell types, such as bacteria, yeast, insects, and mammals [113]. Second method, advances in synthetic peptide chemistry allow scientists to design custom versions of venom toxins, which are more stable, easier to produce, and potentially more effective as medicines. Key techniques used include creating cyclic molecular structures, replacing natural chemical bonds with more stable versions, and incorporating artificial amino acids [114]. Additionally, investigations of recombinant-venom biotechnology show that fusion-tag methods (such as thioredoxin, MBP, or disulfide isomerase domains) enhance the solubility and appropriate folding of disulfide-rich peptides, simplifying downstream purification and functional characterization [115]. Together, these approaches address supply-scale issues and structural complexity. The introduction of customized venom-peptide leads into preclinical and clinical testing, ensuring correct disulfide linkage, and scaling manufacture under GMP requirements remain major obstacles. According to a recent assessment on venom-peptide translation, stringent scale-up, repeatability, and regulatory compliance are still required before venom-derived analgesics may be widely utilized, despite technological breakthroughs [116].

As one of the most representative animal venoms, scorpion venom contains an extremely diverse set of bioactive peptides. Scorpion venom peptides are not only ‘venoms’ that immobilize, paralyze, kill, or dissolve prey but also become important candidates for drug development and design.

## 6. Therapeutic Perspectives and Future Directions

The therapeutic potential of scorpion venom is mostly derived from a variety of low-molecular-weight peptides, often known as scorpion toxins, which are usually between 20 and 90 amino acids long. Neurotoxins are the most common biological activity displayed by these peptides. By altering ion channels in excitable cell membranes, such as sodium, potassium, calcium, and chloride channels, neurotoxic components cause immobilization, paralysis, and eventually the death of prey. When taken as a whole, these processes highlight scorpion venom as an important source of pharmacologically active compounds with substantial therapeutic value [22]. Peptides from scorpion venom have a lot of potential for treating neuropathic pain. This is due to the fact that excessive signaling behind pain is known to be caused by certain sodium channels in injured nerve cells, including NaV1.7, NaV1.8, and NaV1.9. Many scorpion peptides specifically target these channels [17,94]. Furthermore, neuronal hyperexcitability diseases, including epilepsy and neuropathic pain are linked to potassium channel dysfunction or downregulation. Membrane hyperpolarization brought on by potassium channel opening reduces cellular excitability. As a result, several potassium channels, especially those in the Kv1 and Kv7 families, show promise as therapeutic targets for neuropathic pain [24,88]. Makatoxin-3 and ANEP are two of the analgesic peptides from scorpion venom that specifically target sodium channels. These substances, which are derived from *Olivierus martensii* (formerly *Olivierus martensii* Karsch, often designated BMK) and include DKK-SP2, BmKBTx, and BmNaL-3SS2, mostly reduce acute inflammatory pain by obstructing the NaV1.7 channel [18,61,117,118,119,120,121].

Peptides from scorpion venom are useful natural compounds for researching the structure and operation of ion channels, particularly potassium channels. The development of more potent and targeted therapies for autoimmune conditions such as rheumatoid arthritis, multiple sclerosis, type I diabetes mellitus, and systemic lupus erythematosus may be based on compounds that block the Kv1.3 channel [22]. Recombinant ImKTX58 made via genetic engineering techniques has a very selective inhibitory impact on the KV1.3 channel, according to a 2022 study that used electrophysiological measurements [122]. These peptides may be broadly classified into two groups: those with three disulfide bridges and those with four disulfide bridges. By acting on the Kv1.3 channel, these peptides consistently depress the immune system [22]. 

The venom of scorpions is a rich source of bioactive chemicals. Its generated peptides have the potential to be novel cancer therapeutics since they can stop tumor development and spread [123,124]. Anticancer peptides from scorpion venom are classified as either channel-related or non-channel-related based on their molecular structure and target. More than six of these peptides predominantly function by interacting with ion channels such as sodium, potassium, and chloride, and more than thirteen have demonstrated strong benefits against different types of cancer [22]. Emerging preclinical studies suggest that some scorpion-derived compounds could offer dual benefits against cancer pain. They appear to reduce pain signals linked to tumors and alter the tumor environment itself, providing both pain relief and anticancer effects in experimental models [125,126]. These substances have complicated anticancer properties that include several mechanisms, including stopping the development of cancer cells, stopping their dissemination, and inducing cell death. For instance, the scorpion venom peptide BmK AGAP reduces pain by decreasing sodium channel activity in sensory neurons. This reduces neuronal excitability and blocks the transmission of pain signals [24,94].

Using scorpion venom peptides effectively in medicine depends on two essential methods. The first is to create natural peptides that are more selective and stable. These altered molecules become far more resistant to the body’s breakdown by employing strategies including forming stiff peptide structures, substituting disulfide links, and adding synthetic amino acids. As a result, they become more stable and more focused on particular ion channel targets. These modified peptides can retain their original therapeutic benefits while providing improved drug-like qualities and being simpler to create in large quantities, according to a recent study [26,127]. Second method, venom-derived peptides can be made safer and more effective by using tailored delivery methods. The medicine can concentrate where it is required while minimizing exposure to the rest of the body by employing strategies including binding peptides to nanoparticles, encasing them in ligand-directed liposomes, or employing fluorescently labeled peptide probes for specific tissue targeting. These developments enable the delivery of medication directly to afflicted regions, such as injured peripheral nerves or inflamed arthritic joints, and help raise the therapeutic indexes, which results in improved pain relief with fewer side effects [128,129].

Following positive results from lab and animal studies, the next important step is to advance the lab-made venom into Phase 1 clinical trials. These first human trials must be carefully planned and supported by comprehensive safety data, particularly focusing on immunogenic risk, the central nervous system, and cardiovascular system effects [26]. According to clinical evaluations on venom peptide development, early human trials are possible. However, regulatory frameworks highlight that comprehensive preclinical testing and consistent efficacy assessments are crucial for patient safety and therapeutic efficacy before human usage [127].

Natural venom peptides are initial templates that usually require significant improvement. An interdisciplinary effort is crucial to solve problems such as limited specificity, poor stability, and excessive toxicity [24]. The main goal is to use computer models and rapid screening to find versions that bind more strongly to human targets and improve metabolic stability and plasma half-life by applying chemical stabilization methods. Research should also find an effective delivery method for the intended impact on a specific target [92]. Another issue is the incompatibility between results in animals and outcomes in humans. Studies should investigate using animal models that imitate human chronic pain conditions, such as neuropathic, osteoarthritic, or cancer pain, instead of relying only on standard short-term pain tests [24]. In conclusion, creating successful treatments from venom peptides depends on interdisciplinary collaboration. Collaboration across toxin biology, chemical engineering, neurology, and clinical research is crucial to solving today’s issues. By combining these domains, scorpion venom peptides might evolve from intriguing natural venoms into a new class of customized, non-opioid analgesics.

## 7. Discussion

Scorpion venom peptides emerge from this review as structurally diverse, low-molecular-mass molecules that form the main medically relevant fraction of scorpion venoms [22]. Many of these peptides display analgesic, anti-inflammatory, immunomodulatory and anticancer activities in experimental systems [24]. AGAP from *Olivierus martensii* (formerly *Olivierus martensii* Karsch, often designated BMK) reduces somatic and visceral pain in rodent inflammatory and visceral pain models [59]. Makatoxin-3 elicits non-narcotic analgesia in inflammatory pain models such as the formalin and CFA tests [61]. Syb-prII-1 produces strong antinociception in trigeminal neuralgia models while modulating NaV1.8 and MAPK signaling [49]. DKK-SP1 and DKK-SP2 combine anti-inflammatory effects with analgesia via inhibition of NaV1.8 and NaV1.7, respectively [18]. Mechanistically, many scorpion peptides modulate pain-relevant NaV1.7/NaV1.8 and TRP channels as well as other ion channels implicated in nociception [41,94]. AGAP, for example, inhibits TRPV1 and KCNQ2/3 currents and spinal MAPK pathways and can extend the duration of lidocaine analgesia, reducing the required local-anesthetic dose in neuropathic models [19,60]. BmK AGAP also exemplifies dual anticancer and analgesic actions through effects on tumor and sensory-neuron ion channels [22]. These lead peptides and their validated targets are summarized in Table 1, Table 2 and Table 3, and key in vivo studies are highlighted in Table 4.

Despite this compelling preclinical profile, no purified scorpion venom peptide has yet entered randomized controlled trials for human analgesia [86]. Human evidence remains largely restricted to ethnopharmacological reports and case-based observations involving crude venoms with heterogeneous dosing and outcome measures [85]. In this context, translational guidance comes from other venom-derived therapeutics already in clinical use, especially ziconotide (ω-conotoxin MVIIA), an N-type Ca^2+^ channel blocker approved for intrathecal treatment of refractory chronic pain that combines strong analgesia with a narrow therapeutic window and significant neuropsychiatric adverse effects [89]. Broader reviews of venom-based therapeutics emphasize a practical point: whether these drugs succeed often comes down to peptide selectivity, the route of administration, and careful pharmacokinetic optimization [113]. A similar issue appears with selective sodium-channel blockers. Despite strong preclinical promise, several NaV1.7 inhibitors—including PF-05089771 and CNV1014802—produced disappointing or inconsistent results in clinical pain trials [95]. In the same vein, the NaV1.8 inhibitor VX-548 showed clear benefit in acute pain, but delivered mixed outcomes in phase II sciatica studies, which tempers expectations that targeting peripheral sodium channels will be universally effective [97]. At the same time, some scorpion toxins such as BmK I are pronociceptive, enhancing NaV1.8 currents and driving spontaneous pain and mechanical allodynia in rodent models [50]. Whole-venom studies in *Tityus serrulatus* also show robust hyperalgesia mediated by TRPV1 activation, immune-cell recruitment and pro-inflammatory cytokines, underscoring that not all venom components are therapeutically desirable [109].

Preclinical safety data suggest that isolated venom peptides can achieve useful therapeutic windows when separated from toxic crude components, with several peptides showing in vivo analgesic or anti-inflammatory effects without overt organ damage or behavioral toxicity at tested doses [17]. Buthicyclin is a recent example of a venom-derived peptide with significant analgesic activity and an LD_50_ > 20 mg/kg in rodents, indicating low acute toxicity within its effective range [99]. Nevertheless, envenomation studies highlight that scorpion toxins can affect cardiac and central targets and trigger severe systemic manifestations, so off-target channel interactions remain a major translational concern [110]. Venom composition varies with species, geography and extraction methods, and proteomic work shows that this variability complicates pharmacological reproducibility and demands standardized workflows for collection, fractionation and folding verification [105]. Moving from crude venom to recombinant or synthetic peptides with defined sequence and structure is therefore essential for GMP-grade manufacturing and regulatory approval [26]. The current evidence base is dominated by rodent models using acute or subchronic endpoints, and there are still no randomized controlled trials of purified scorpion peptides for pain, in contrast to conotoxins and other venom-derived drugs [86]. Reviews on venom-peptide development stress that first-in-human studies must prioritize immunogenicity, cardiovascular and CNS safety while integrating robust PK/PD and standardized pain outcomes [127]. The present review is further limited by its reliance on English-language and indexed literature, which may under-represent regional and non-English data on scorpion-based analgesic practices. Future discovery efforts should prioritize understudied scorpion families and non-NaV pain mechanisms, including neuroimmune interactions and central synaptic plasticity, to expand the translational pipeline beyond currently favored targets.

## 8. Conclusions

Peptides isolated from scorpion venom emerge as a promising yet underexplored avenue for opioid-independent analgesia. Diverse in structure, these peptides interact with multiple molecular targets, thereby regulating ion channels and suppressing inflammation. Animal experiments confirm their utility. Rodent models of acute, inflammatory, and neuropathic pain show relief levels akin to morphine, absent the addiction profile. Safety assessments reveal manageable toxicity when dosages are calibrated precisely. Obstacles to clinical application persist, including venom batch variability, risks of immune activation, and sparse human evidence. Recombinant production addresses some issues. Standardization is needed. Comprehensive trials should follow. Future strategies may incorporate artificial intelligence for peptide optimization. Dual functionalities stand out: analgesia coupled with antitumor actions in compounds like BmK AGAP. Multicenter collaborations could advance testing. Efforts center on exploiting these venom-derived agents against chronic pain, potentially altering paradigms in the opioid crisis.

## 9. Materials and Methods

We conducted a systematic search of the literature for this narrative review using PubMed, Scopus and Web of Science. The keywords were ‘scorpion venom peptides’ AND (‘pain relief’ OR ‘analgesic’ OR ‘pain management’ OR ‘antinociceptive’); we also included variations such as ‘Olivierus martensii peptides’ or ‘Tityus serrulatus toxins’. Publications ranged from January 2000 through November 2025. That captured new developments along with some background.

We included peer-reviewed articles published in English. Priority was given to reviews and primary studies reporting analgesic/antinociceptive activity in animal or human models and to mechanistic studies relevant to pain modulation, particularly those involving ion channels and inflammatory signaling. We excluded non–peer-reviewed literature and studies focused solely on antimicrobial, cytotoxic, or other non-pain indications without a plausible link to pain pathways or outcomes.

To improve coverage, we hand-searched the reference lists of key reviews and highly relevant primary papers. Supplementary manual screening of references from key reviews ensured comprehensiveness [22]. Final inclusion decisions were agreed upon by the authors to ensure full coverage. The search retrieved about 115 relevant publications, with studies published between 2020 and 2025 providing the most recent developments.

The generative AI tool Google Gemini was used only to assist in drafting and refining schematic figures; it was not used to generate scientific claims, extract data, or perform analyses.

## Figures and Tables

**Table 1:** Key scorpion venom peptides with analgesic potential.

Peptide	Source Species	Target	Pain Type Addressed	Key Studies/Effects
BmK AGAP	*Buthus martensii* Karsch	HV1; TRPV1; KCNQ2/3; NaV; spinal MAPKs	Inflammatory; visceral; neuropathic (co-admin with lidocaine)	Reduces somatic/visceral pain; inhibits spinal MAPKs; potentiates/extends lidocaine analgesia [19,22,59,60]
Syb-prII-1	Scorpion neurotoxin (β-NaTx)	NaV1.8; MAPK pathway	Trigeminal neuralgia (IoN-CCI)	Morphine-comparable antinociception; acts via NaV1.8 and MAPKs [49]
Makatoxin-3	*Buthus martensii* Karsch	NaV1.7	Inflammatory (formalin, CFA)	Non-narcotic analgesia in formalin/CFA models [61]
BmK I (pronociceptive control/toxicity context)	*Buthus martensii* Karsch	NaV1.8	Sting pain/inflammatory hypersensitivity	Increases NaV1.8 current/excitability; induces spontaneous pain and allodynia; effects reduced by NaV1.8 block/knockdown [50]
SVHRP (and synthetic derivative SVHRSP)	*Buthus martensii* Karsch	Microglia; NF-κB/MAPK signaling	Neuroinflammatory models	Reduces microglial activation and pro-inflammatory mediators (evidence mainly acute paradigms); SVHRSP shows neuroprotective anti-neuroinflammatory effects in disease/toxin models [54,55,56,57,58]
DKK-SP1/2	*Buthus martensii* Karsch	DKK-SP1: NaV1.8; DKK-SP2: NaV1.7	Anti-inflammatory + analgesic effects across rodent pain/inflammation models	Anti-inflammatory + analgesia via NaV inhibition (DKK-SP1 ↓NaV1.8 expression/current and shifts cytokines; DKK-SP2 inhibits hNaV1.7 expression/current) [18]
TsNTxP	*Tityus serrulatus*	Suppresses presynaptic glutamate release	Antinociception in mouse models	Antinociceptive effects via glutamate-release suppression [21]

Abbreviations: HV1, voltage-gated proton channel 1; TRPV1, transient receptor potential vanilloid 1; KCNQ2/3 (Kv7.2/Kv7.3), voltage-gated potassium channels KCNQ2 and KCNQ3; NaV, voltage-gated sodium channel; NaV1.7/NaV1.8, voltage-gated sodium channel subtypes 1.7/1.8; MAPK(s), mitogen-activated protein kinase(s); NF-κB, nuclear factor kappa B; CFA, Complete Freund’s Adjuvant; IoN-CCI, infraorbital nerve chronic constriction injury; β-NaTx, beta sodium-channel toxin; SVHRP, scorpion venom heat-resistant peptide; SVHRSP, synthetic derivative of SVHRP.

**Table 2:** Structural and Functional Classification of Analgesic Scorpion Venom Peptides.

Peptide	Species (Taxonomy)	Family	DBP/NDBP	Primary Target(s)	Key Analgesic Effect	Reference(s)
BmK-AGAP	*Olivierus martensii*	Buthidae	DBP	NaV, TRPV1, KCNQ2/3	Neuropathic and inflammatory analgesia	[19,59,60,62]
IMe-AGAP	*Mesobuthus eupeus*	Buthidae	DBP	NaV	Antitumor–analgesic activity	[35]
Makatoxin-3	*Olivierus martensii*	Buthidae	DBP	NaV1.7	Non-opioid inflammatory pain relief	[61]
TsNTxP	*Tityus serrulatus*	Buthidae	Protein	Glutamate release	Neuropathic pain reduction	[21]

**Table 3:** Sources, Experimental Models, and Translational Status.

**Peptide**	**Experimental Model(s)**	**Dose Range**	**Comparator**	**Translational Status**	**Reference(s)**
BmK-AGAP	CCI, formalin	25–100 μg/kg	Morphine, lidocaine	Preclinical	[19,59,60]
Syb-prII-1	IoN-CCI	~4 mg/kg	Morphine	Preclinical	[49]
Makatoxin-3	Formalin, CFA	~450 nmol/kg	NSAIDs	Preclinical	[61]
TsNTxP	Tail-flick, capsaicin	μg/kg range	Carbamazepine	Preclinical	[21]

**Table 4:** Preclinical Evidence.

Study/Year	Model	Findings	Limitations	References
Bai et al., 2022	IoN-CCI (trigeminal neuralgia)	Syb-prII-1: Morphine-comparable analgesia; NaV1.8 downregulation	Rodent-specific; no long-term data	[49]
Chen et al., 2022	Formalin, CFA	Makatoxin-3: Potent non-narcotic relief	Acute/inflammatory focus; translation gaps	[61]
Kampo et al., 2021	CCI + lidocaine co-administration	BmK AGAP: 50% ED50 reduction; synergy	Synergy model-specific; species differences	[19]
Rigo et al., 2019	Various (glutamate suppression)	TsNTxP: Antinociception without toxicity	Preclinical only; mechanism indirect	[21]
Liu et al., 2021	Acetic acid, formalin	DKK-SP1/2: Anti-inflammatory analgesia	Limited to BmK; no human extrapolation	[18]

Abbreviations: IoN-CCI, infraorbital nerve chronic constriction injury; CFA, Complete Freund’s Adjuvant; CCI, chronic constriction injury; ED50, median effective dose (50% effective dose).

## Data Availability

No new data were created or analyzed in this study.
